# HIV-1 Nef-induced lncRNA AK006025 regulates CXCL9/10/11 cluster gene expression in astrocytes through interaction with CBP/P300

**DOI:** 10.1186/s12974-018-1343-x

**Published:** 2018-10-31

**Authors:** Feng Zhou, Xiaomei Liu, Dongjiao Zuo, Min Xue, Lin Gao, Ying Yang, Jing Wang, Liping Niu, Qianwen Cao, Xiangyang Li, Hui Hua, Bo Zhang, Minmin Hu, Dianshuai Gao, Kuiyang Zheng, Yoshihiro Izumiya, Renxian Tang

**Affiliations:** 1 Jiangsu Key Laboratory of Brain Disease Bioinformation, Research Center for Biochemistry and Molecular Biology, Xuzhou Medical University, Xuzhou, 221004 Jiangsu People’s Republic of China; 2 Jiangsu Key Laboratory of Immunity and Metabolism, Department of Pathogen Biology and Immunology and Laboratory of Infection and Immunity, Xuzhou Medical University, Xuzhou, 221004 Jiangsu People’s Republic of China; 3 Department of Physiology, Xuzhou Medical University, Xuzhou, 221004 Jiangsu People’s Republic of China; 4 Department of Neurobiology and Anatomy, Xuzhou Medical University, Xuzhou, 221004 Jiangsu People’s Republic of China; 5 Department of Dermatology, University of California Davis (UC Davis) School of Medicine, Sacramento, CA USA

**Keywords:** HIV-associated neurocognitive disorder, HIV-1, lncRNAs, Astrocytes, Nef, CBP/P300, Inflammation

## Abstract

**Background:**

HIV-associated neurocognitive disorder (HAND) is a neurodegenerative disease associated with persistent neuroinflammation and subsequent neuron damage. Pro-inflammatory factors and neurotoxins from activated astrocytes by HIV-1 itself and its encoded proteins, including the negative factor (Nef), are involved in the pathogenesis of HAND. This study was designed to find potential lncRNAs that regulate astrocyte functions and inflammation process.

**Methods:**

We performed microarray analysis of lncRNAs from primary mouse astrocytes treated with Nef protein. Top ten lncRNAs were validated through real-time PCR analysis. Gene ontology (GO) and KEGG pathway analysis were applied to explore the potential functions of lncRNAs. RIP and ChIP assays were performed to demonstrate the mechanism of lncRNA regulating gene expression.

**Results:**

There were 638 co-upregulated lncRNAs and 372 co-downregulated lncRNAs in primary astrocytes treated with Nef protein for both 6 h and 12 h. GO and KEGG pathway analysis showed that the biological functions of top differential-expressed mRNAs were associated with inflammatory cytokines and chemokine. Knockdown of lncRNA AK006025, not AK138360, inhibited significantly CXCL9, CXCL10 (IP-10), and CXCL11 expression in astrocytes treated with Nef protein. Mechanism study showed that AK006025 associated with CBP/P300 was enriched in the promoter of CXCL9, CXCL10, and CXCL11 genes.

**Conclusions:**

Our findings uncovered the expression profiles of lncRNAs and mRNAs in vitro, which might help to understand the pathways that regulate astrocyte activation during the process of HAND.

**Electronic supplementary material:**

The online version of this article (10.1186/s12974-018-1343-x) contains supplementary material, which is available to authorized users.

## Background

Human immunodeficiency virus type 1 (HIV-1) infection has been demonstrated to be associated with HIV-associated neurocognitive disorders (HAND) that is characterized by degenerative loss of neurons and ultimately develops into HIV-associated dementia (HAD), the most severe form of HAND [1, 2]. Despite the use of highly active antiretroviral therapy (HAART) has successfully prevented many of the former end-stage complications of acquired immunodeficiency syndrome (AIDS), neuronal cell death remains a problem that is frequently found in the brains of HIV-1-infected patients. With prolonged survival of AIDS patients, the prevalence of minor cognitive motor disorder appears to be rising among AIDS patients. Furthermore, HAD is still prevalent in treated AIDS patients.

HAND pathogenesis is associated with the direct and indirect effects of HIV-1 infection on the neuron damage and loss [3]. The direct effects from HIV-1 infection in the central nervous system (CNS) are from the neurotoxicity of HIV-1 itself [4–6] and HIV-1-encoded proteins including gp120 [7–10], transactivator of transcription (Tat) [3, 11–14], and negative factor (Nef) [15, 16], whereas the indirect toxicity is due to altered glutamate neurotransmission [5], as well as pro-inflammatory cytokines that are secreted by astrocytes or microglia infected with HIV-1 or stimulated with HIV-1 proteins [3, 17].

Astrocytes, the major glial cell type within the CNS [18], support neuron function through promoting formation and function of synapses and build the formation of the blood-brain barrier (BBB) [19]. Furthermore, astrocytes possess the capacity to interact with the peripheral immune system by recruiting leukocytes and monocytes into the CNS [20]. Reactive astrocytosis is an important feature in an inflammatory condition that occurs during HAND pathology [21]. Experimental data indicate that astrocyte activation is involved in the pathogenesis of HAND characterized by an increased expression of pro-inflammatory cytokines and chemokine [3, 17]. In response to HIV-1 and its encoded protein, Nef, in the CNS, astrocytes increase chemokine production to facilitate T cells and monocytes recruitment to the CNS [17]. However, the mechanisms by which astrocyte activation contribute to HAND by Nef remain still unknown.

Nef is a 27-kDa myristoylated protein released from HIV-infected cells in the exosome manner [22–24]. Nef interacts with a multitude of cellular factors and functions in trafficking, signal transduction, and gene expression. Nef leads to neural cell death directly and indirectly through the production of CXCL10 (IP-10) [25]. Functionally, Nef expressed in astrocytes results in impairing spatial and recognition memory in rats [26]. Clinical data display that Nef expresses in hippocampal neurons in HIV^+^ patients with HAD [27], consistent with this protein being involved in the memory impairment of those individuals. Of important, HIV-1 Nef induces CCL5 production in astrocytes to promote neuron death through p38-MAPK and PI3K/Akt pathway and utilization of transcription factors, such as NF-κB, C/EBP, and AP-1 [16]. These exciting results have prompted us to scan the expression profiles of inflammatory cytokines and chemokine in astrocytes induced by Nef and their roles in HAND pathogenesis.

Long non-coding RNAs (lncRNAs) have been defined as RNA transcripts more than 200 nucleotides in length that do not encode proteins. Based on the lncRNA-related position in the genome to the protein-coding genes, lncRNAs include five categories, which are sense, antisense, intronic, intergenic, and bidirectional [28]. Emerging evidence suggests that they play key roles in various biological and physiological processes including chromatin remodeling, epigenetic regulation, RNA splicing, and protein transport and directly relate to human diseases including neurodegenerative disorders [29–32]. One of the major functions of lncRNAs appears to be in the epigenetic regulation of gene transcription [32]. For example, lncRNAs have been shown to associate with a plethora of epigenetic modifier complexes including PRC2, PRC1, Cbx1, Cbx3, Tip60/P400, Setd8, ESET and Suv39h1, Jarid1b, Jarid1c, HDAC1, and YY1 [33, 34]. It has been reported that lncRNA H19 promotes neuroinflammation in ischemic stroke by driving HDAC1-dependent M1 microglial polarization [35]. In addition, lncRNAs activate NF-κB and MAP kinase pathway to regulate inflammation gene expression [36–39]. However, the roles of lncRNAs in the process of HAND are still unknown.

CREB-binding protein (CBP)/P300, histone acetyltransferase (HAT), is transcription co-activator that is *cis*-regulatory elements to control patterns of gene expression. By the enzymatic activity localized in HAT domain, CBP/P300 target both histones and numerous transcriptional factors (TFs), leading to elevated histone 3 lysine 27 acetylation (H3K27ac), p53, NF-κB, and RNA Pol II acetylation that forms a transcription network “hub” to promote gene expression [40–45]. Recent data show that CBP/P300 binds directly to enhancer RNAs (eRNAs) to stimulate histone acetylation and gene transcription [46]. Moreover, lncRNAs bind CBP/P300 activity to regulate gene expression [47].

In the present study, we analyzed the lncRNA and mRNA expression landscape of astrocytes treated by Nef protein in vitro. Our results suggest potential roles of lncRNAs in regulating inflammation in astrocytes during the process of HAND.

## Methods

### Animals

C57BL/6 mice were obtained from Nanjing University Laboratory Animal Center. Mice were housed at 25 ± 1 °C with 55–65% humidity and maintained under specific pathogen-free conditions. All experimental procedures described in our study were performed according to the Provision and General Recommendation of the Chinese Laboratory Association. The protocol was approved by the Institutional Animal Care and Use Committee of Xuzhou Medical University.

### Primary astrocyte culture

Primary mouse astrocytes from 0- to 1-day-old C57BL/6 mice were established as previously described [48]. In brief, the cerebral cortices freed of meninges were dissected, minced, and digested. After washed twice in DMEM/F12 medium containing 10% fetal bovine serum (FBS), the cells were filtrated, transferred to culture flasks pre-coated with 1 mg/ml poly-L-lysine (Sigma), and then cultured at 37 °C with 5% CO_2_. Cells were passed for three passages and then glial fibrillary acidic protein (GFAP, astrocytic marker) expression was analyzed using immunofluorescence assays (IFA). Finally, at least 95% GAFP^+^ cells were used to research.

Primary human astrocytes were obtained from ScienCell Research Laboratories with the help of Shanghai Zhong Qiao Xin Zhou Biotechnology Co., Ltd. in China. Human astrocytes were cultured in the Astrocyte Medium (AM, Cat. No. 1801) contained 2% FBS (Cat. No. 0010) and astrocyte growth supplement (AGS, Cat. No. 1852) at 37 °C with 5% CO_2_. Cells were passed for three passages and performed to research.

### RNA extraction

Total RNA was extracted from the primary astrocytes according to manufactures instructions. RNA quantity and quality was measured using NanoDrop ND-1000 spectrophotometer (Thermo Fisher Scientific).

### Determination of lncRNA and mRNA profiles

The expression profiles of lncRNAs and mRNAs were detected as previously described [49]. Primary mouse astrocytes treated with 50 ng/ml Nef protein (Abcam, ab90462) were detected using Mouse LncRNA Microarray v2.0 (8 × 60 K, Arraystar) by Kangchen Bio-tech (Shanghai, China) that designed for the global profiling of mouse LncRNAs and protein-coding transcripts. Thirty-one thousand four hundred twenty-three LncRNAs and 25,376 coding transcripts can be detected by the second-generation LncRNA microarray. The LncRNAs are carefully collected from the most authoritative databases such as RefSeq, UCSC Known Genes, Ensembl, and many related literatures. Each transcript is represented by a specific exon or splice junction probe which can identify accurately individual transcript. Positive probes for housekeeping genes and negative probes are also printed onto the array as hybridization quality control.

According to previous experimental project, microarray assays had also been performed using pooled plasma, blood, liver, heart, or cell samples [50]. RNAs from mixed astrocytes treated with Nef protein three times were run microarray analysis. The acquired raw array images were processed by Agilent Feature Extraction software (version 11.0.1.1) and then normalized and analyzed by the GeneSpring GX v12.0 software package (Agilent Technologies). Differentially expressed lncRNAs and mRNAs were then identified through fold change as well as *P* values calculated with *t* test. The threshold for up and downregulation was fold change > 2.0 and *P* value < 0.05. Afterwards, hierarchical clustering was performed to display the distinguishable lncRNA and mRNA expression patterns among the samples.

### Real-time PCR assay

Real-time PCR (real-time PCR) assay was detected as previously described [49]. In brief, the total RNA from cultured astrocytes was extracted with TRIzol reagent (Invitrogen). First-strand cDNAs were generated using PrimeScriptTM RT reagent kit (TaKaRa, Japan), and SYBR Premix Ex TaqTM based on real-time PCR (TaKaRa) was performed to analyze the relative expression levels of lncRNAs and mRNAs. The relative gene expression was calculated using the 2 ^−ΔΔ*CT*^ method. The primers are listed in Additional file 1: Table S1.

### Functional group analysis of mRNAs

Gene ontology (GO) and KEGG analysis were applied to determine the functions of differentially expressed mRNAs in biological pathways using the standard enrichment computation method [49]. The *P* value (hypergeometric *P* value) denotes the significance of the pathway correlated to the conditions. The recommend *P* value cutoff is 0.05.

### ELISA

The levels of Cxcl9, Cxcl10, and Cxcl11 in the supernatants of cultured astrocytes were detected using Mouse ELISA kits (Abcam) according to the manufacturer’s instructions.

### RNA immunoprecipitation (RIP) assay

RIP assay was performed as previously described [51].In brief, after treated with Nef protein, 2 × 10^7^ primary mouse astrocytes were treated with 0.3% formaldehyde in DMEM/F12 medium for 10 min at 37 °C. Glycine dissolved in PBS (1.25 M) was added to the final concentration of 0.125 M for 5 min at room temperature (RT). Cells were then washed twice in cold PBS and pelleted. The pellets were resuspended in RIPA buffer and incubated on ice with frequent vortex for 30 min, and the lysate was centrifugated at 13,000 RPM for 10 min. Antibodies (5 μg) were added and incubated for 4 h at 4 °C. Samples were washed twice in RIPA buffer, four times in 1 M RIPA buffer, and then twice in RIPA in spin columns. The beads were resuspended in RIPA buffer and treated with proteinase K at 45 °C for 45 min. RNA samples were extracted with Trizol. Co-precipitated RNAs were purified with the RNeasy Mini Kit (QIAGEN) and detected by real-time PCR. The data of retrieved RNAs is calculated from the subtraction of RT/input ratio and non-RT/input ratio. The primer sequence was listed in Additional file 1: Table S1.

### Chromatin immunoprecipitation (ChIP) assay

ChIP assays were performed as described previously [52]. Briefly, primary mouse astrocytes were treated with or without Nef protein for 3 h. Cells were cross-linked with 1% formaldehyde (final concentration) for 10 min at RT and then stopped by 1.25 mM of glycine (final concentration). 1 × 10^7^ cells were used for each ChIP-enrichment. Chromatin was sheared to the fragment size of 200–500 bp using a Bioruptor (Diagenode). The antibodies used in the ChIP experiments were anti-NF-κB p65 antibody (Cell Signaling Technologies), anti-H3K27ac antibody (Abcam), anti-RNA Pol II antibody (Active Motif), anti-CBP/P300 antibody (Cell Signaling Technologies), normal mouse IgG (Santa Cruz). All immunoprecipitated chromatin DNA was analyzed by real-time PCR. The primer sequences were listed in Additional file 1: Table S1.

### Knockdown of lncRNAs

To silence the mouse lncRNA AK006025 and AK138360, siRNA sequences against AK006025 and AK138360 were designed. siRNA sequences were as follows: si-Ctrl, 5′-UUC UCC GAA CGU GUC ACG UTT-3′; si-AK006025, 5′-GCT GGG AAC CCA CAC ATA A-3′ and si-AK138360, 5′-GCA CCG GGC AAT GTT TAA T-3′.

### Statistical analysis

SPSS version 18.0 was used for the statistical analysis of experimental data. Data presented as mean ± standard error of mean (SEM), calculated for all points from at least three independent experiments in triplicates. Statistical significance was determined using the two-tailed Student’s *t* test or by one-way analysis of variance (ANOVA) when more than two groups were compared. *P* < 0.05 was considered significant.

## Results

### Differentially expressed mRNA in the primary mouse astrocytes treated with Nef protein

**Fig. 1:**
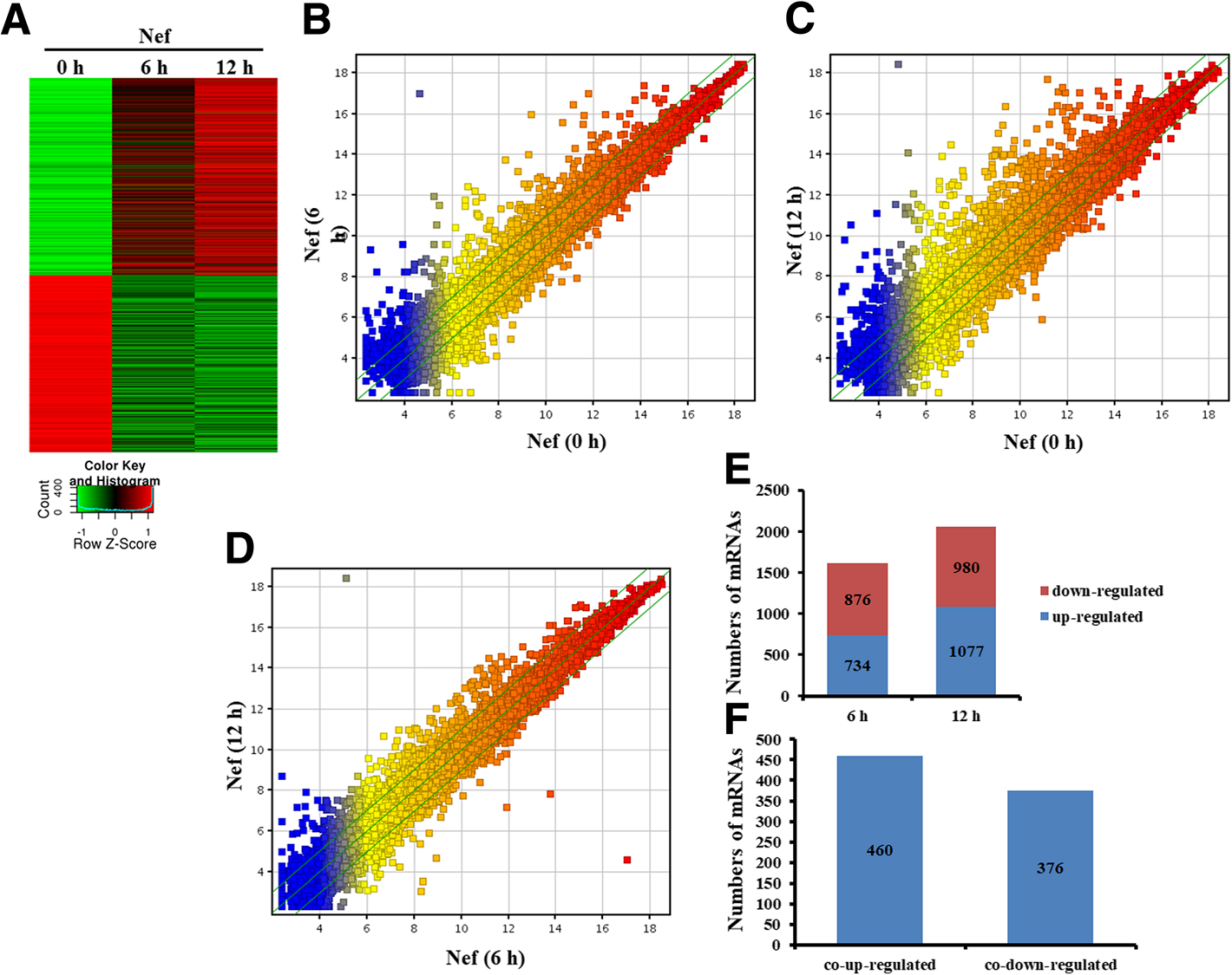
Identification of differentially expressed mRNAs in primary mouse astrocytes treated with Nef protein. **a** Heatmap of mRNAs significantly changed upon Nef (50 ng/ml) stimulation of astrocytes. **b**, **c**, **d** Scatter plot compared global mRNA gene expression profiles in Nef-treated astrocytes. The values of X-axis and Y-axis in the scatter plot were the normalized signal values of each sample (log2 scaled). The green lines are fold change (the default fold change value given is 2.0). The mRNAs above the top green line and below the bottom green line indicate more than 2.0-fold change, compared to the control. **e** Analysis of numbers of significantly expressed mRNAs. **f** Overlapped and differentially expressed mRNAs

To detect differentially expressed mRNAs in the activated mouse primary astrocytes, we performed a genome-wide analysis of mRNA expression profiles in the astrocytes stimulated with Nef protein (50 ng/ml) at the different time point (Fig. 1a). Firstly, a graphical overview of the expression signatures of mRNAs was performed by scatter plot analyses. The result displayed that a great number of mRNAs were differentially expressed in the astrocytes with Nef protein at 0 h, 6 h, and 12 h (Fig. 1b, c). Meanwhile, many mRNAs were differentially co-expressed in the astrocytes with Nef protein at 6 h and 12 h (Fig. 1d).

We then analyzed altered expression of mRNAs with fold change > 2, the criteria *P* value < 0.05 in the astrocytes stimulated by Nef protein at 6 h and 12 h compared with 0 h, respectively. Results showed that 734 mRNAs were upregulated, and 876 mRNAs were downregulated in the astrocytes stimulated by Nef protein for 6 h (Fig. 1e) and that 1077 upregulated and 980 downregulated mRNAs for 12 h (Fig. 1e). Notably, there were 460 co-upregulated and 376 co-downregulated mRNAs for between 6 h and 12 h (Fig. 1f). Importantly, there were many inflammatory cytokines and chemokine, especially highly expressed *Cxcl11*, *Cxcl10* (IP-10), *Cxcl9*, *Cxcl3*, *Cxcl2*, *Cxcl1*, *Ccl5*, *Il-1β*, *Il-6*, and *Tnf*, among the co-upregulated mRNAs (Table 1).

**Table 1:** Top 20 overlapped and upregulated mRNAs in mouse astrocytes treated with Nef protein (50 ng/ml) for 6 h and 12 h

Gene symbol	Upregulated folds	
	6 h/0 h	12 h/0 h
Cxcl11	105.05796	473.09506
Cxcl9	43.443104	213.93571
Ifnb1	112.73581	154.63144
Il1b	54.497185	153.78612
Gm4841	52.749744	149.3729
Cxcl2	98.37795	131.17091
Irg1	69.53818	101.9587
Ccl5	39.71899	94.32046
Il6	21.031158	74.925606
Ccl7	27.82146	74.49636
Fam26f	11.689228	64.880646
Cd69	30.492313	53.828453
Cxcl10	37.51984	45.796886
Cxcl1	20.680431	45.20861
Il1a	13.884002	49.921574
Cxcl3	38.239742	43.34039
Tnfsf10	4.3851957	24.53629
Tnf	30.36271	24.412766
Ccl3	11.2570715	15.065439
Ccl2	9.46433	10.722074

**Fig. 2:**
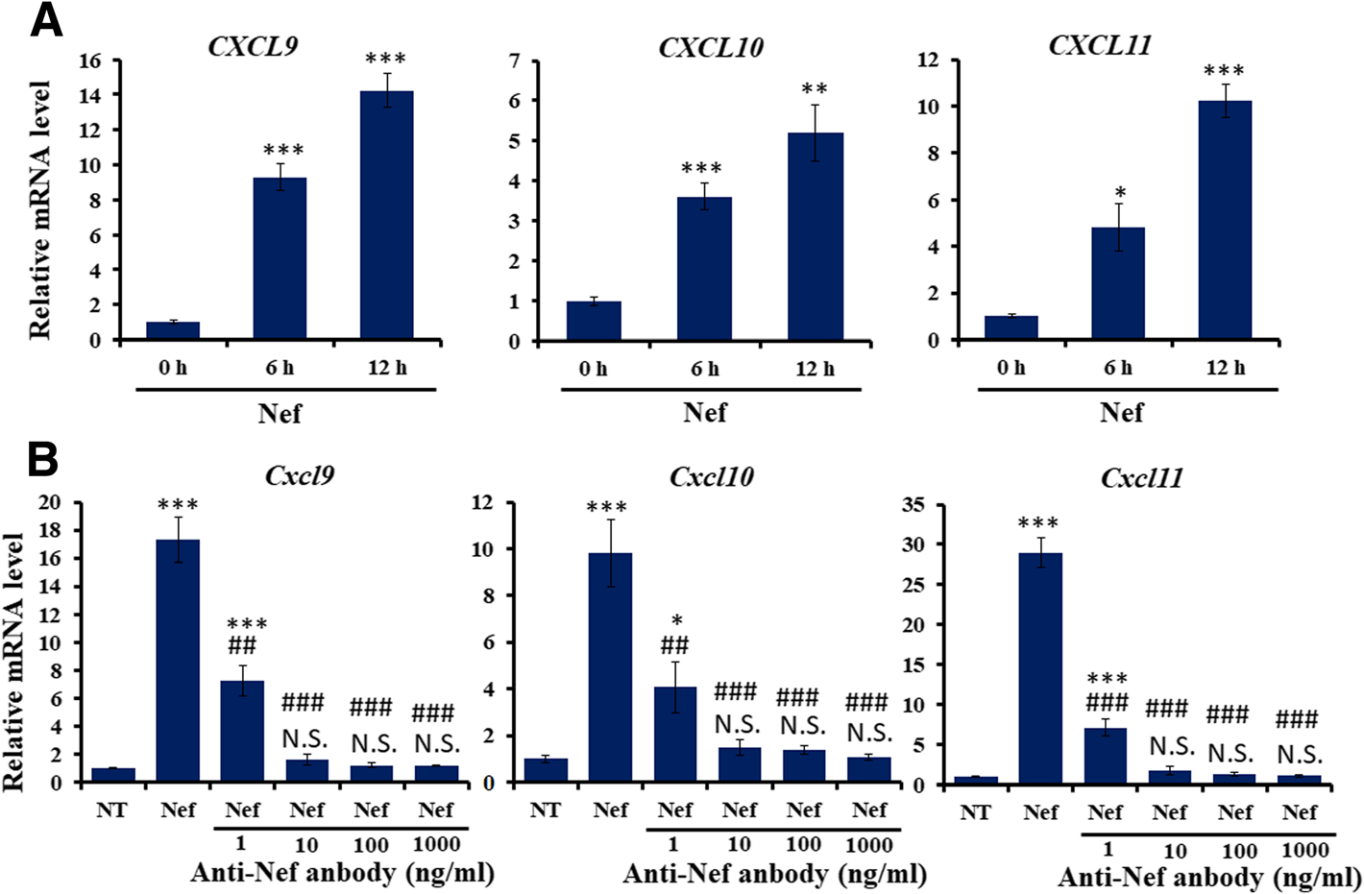
Determination of effects of Nef protein on CXCL9/10/11 gene expression. **a** Primary human astrocytes were treated with soluble Nef protein (50 ng/ml) for 6 h and 12 h. Human *CXCL9*, *CXCL10*, and *CXCL11* mRNA levels were determined by real-time PCR assay (*n* = 3). Error bars represent means ± SEM. *P < 0.05, **P < 0.01, ***P < 0.001, vs 0 h. **b** Primary mouse astrocytes were pre-treated with different concentration of anti-Nef antibody followed by soluble Nef protein (50 ng/ml) for 6 h. Mouse *Cxcl9*, *Cxcl10*, and *Cxcl11* mRNA levels were determined by real-time PCR assay (*n* = 3). Error bars represent means ± SEM. *P < 0.05, ***P < 0.001, vs NT. ^##^*P* < 0.01, ^###^*P* < 0.001, vs Nef

To confirm the role of Nef protein in chemokine expression in human astrocytes, human primary astrocytes were treated with Nef protein for 6 h, *CXCL9*, *CXCL10*, and *CXCL11* mRNA levels were determined by real-time PCR assay. The results showed that mRNA levels of *CXCL9*, *CXCL10*, and *CXCL11* were significantly increased in human astrocytes treated with Nef at 6 h and 12 h (Fig. 2a), which was inconsistent with mouse astrocytes. To further determine upregulated mouse *Cxcl9*, *Cxcl10*, and *Cxcl11* by soluble recombinant Nef protein rather than by other factors like lipopolysaccharide (LPS), mouse primary astrocytes were immunoeluted with Nef (iNef) protein through using anti-Nef antibody (Abcam, ab42355) pretreatment prior to Nef stimulation for 6 h. Real-time PCR assay indicated that iNef did not induce mRNA levels of *Cxcl9*, *Cxcl10*, and *Cxcl11* (Fig. 2b). These results suggest that Nef itself enhances CXCL11, CXCL10, and CXCL9 expression in human and mouse astrocytes.

### Go and KEGG pathway analysis of the molecular function of differentially expressed genes

**Fig. 3:**
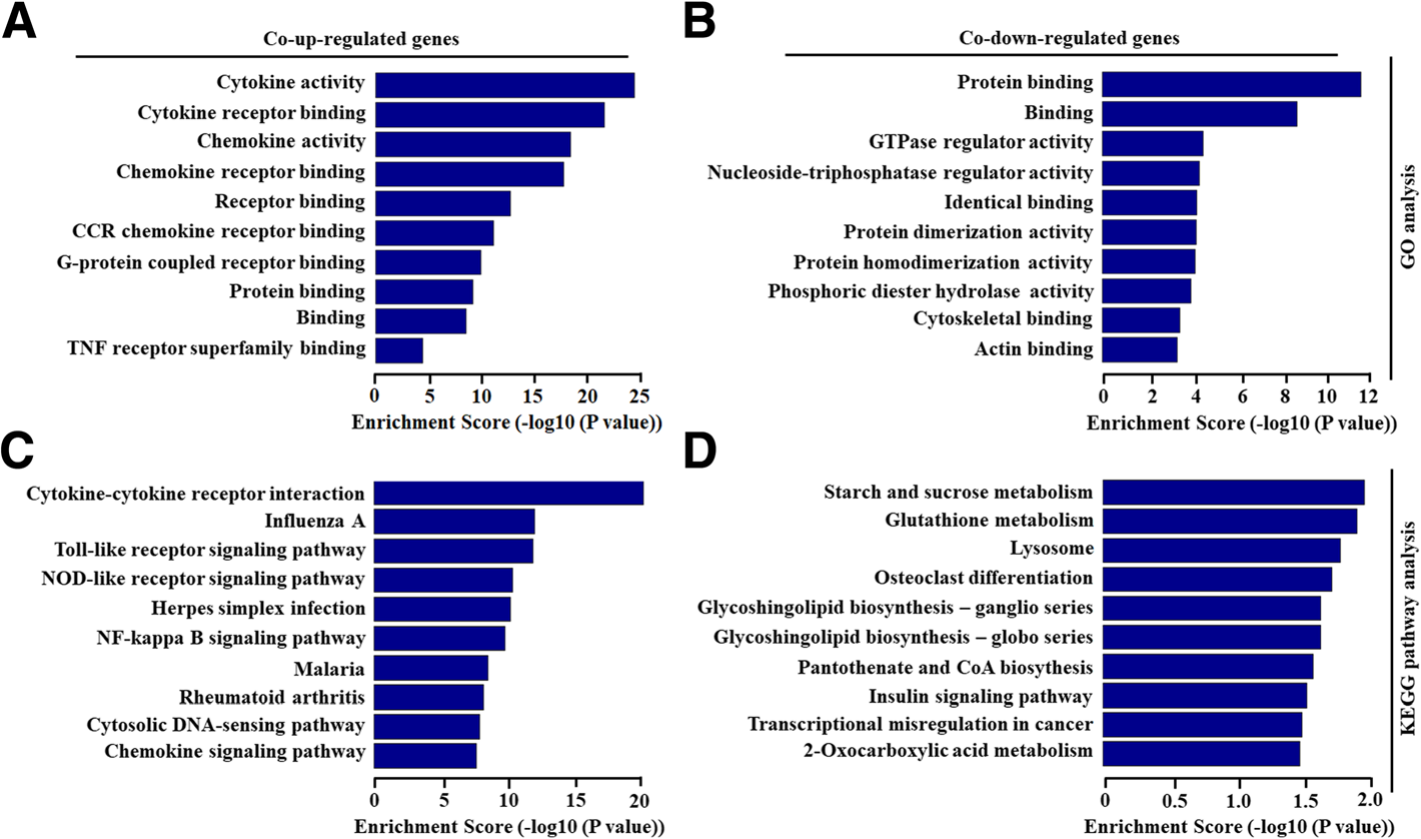
Biological function of differentially overlapped genes with fold changes more than 2.0. **a**, **b** GO analysis of significant molecular function of co-upregulated and co-downregulated genes were shown in primary mouse astrocytes treated by Nef protein (50 ng/ml) with 6 h and 12 h. **c, d** KEGG pathway analysis of significant pathways for co-upregulated and co-downregulated genes were shown in primary mouse astrocytes treated by Nef protein with 6 h and 12 h

To further explore potential molecular mechanism in HAND pathogenesis, GO and KEGG pathway analysis was performed for differentially expressed genes in astrocytes treated with Nef protein. GO analysis showed that the molecular function (MF) of differentially co-upregulated expressed transcripts was associated with cytokine and chemokine activity as well as their receptor binding in astrocytes treated with Nef protein (Fig. 3a). Furthermore, differentially co-downregulated genes were mainly related with protein binding in astrocytes treated with Nef protein (Fig. 3b).

KEGG pathway analysis indicated that 40 pathways were significantly involved in differentially expressed genes. Upregulated genes were enriched in cytokine and its receptor interaction, chemokine signaling pathway, NF-κB signaling pathway, and so on (Fig. 3c). Downregulated genes were involved in metabolism, lysosome, CoA biosynthesis, insulin signaling pathway, etc. (Fig. 3d). The above data represent that HAND is associated with the mRNA changes of inflammatory factors in the astrocytes.

### Differentially expressed lncRNA in the primary mouse astrocytes treated with Nef protein

**Fig. 4:**
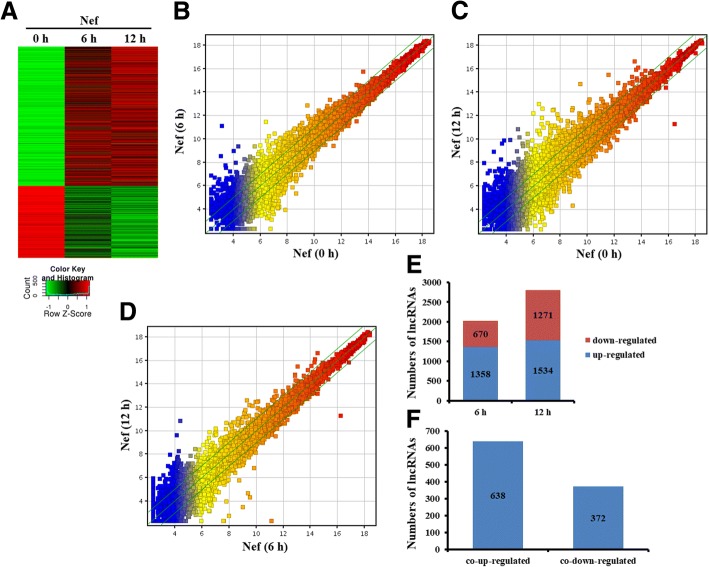
Analysis of differentially expressed lncRNAs in primary mouse astrocytes treated with Nef protein. **a** Heatmap of lincRNAs significantly changed upon Nef (50 ng/ml) stimulation of astrocytes. **b**, **c**, **d** Scatter plot compared global lncRNA expression profiles in Nef-treated astrocytes. The values of X-axis and Y-axis in the scatter plot were the normalized signal values of each sample (log2 scaled). The green lines are fold change (the default fold change value given is 2.0). The mRNAs above the top green line and below the bottom green line indicate more than 2.0-fold change, compared to the control. **e** Analysis of numbers of significantly expressed lncRNAs. **f** Overlapped and differentially expressed lncRNAs

We further determined differentially expressed lncRNAs in the astrocytes treated with Nef protein at the different time point (Fig. 4a). Scatter plot analyses were firstly used to scan the expression signatures of lncRNAs. The result showed that a large number of lncRNAs were differentially expressed in the astrocytes with Nef protein at 0 h, 6 h, and 12 h (Fig. 4b, c). Meanwhile, lots of lncRNAs were differentially expressed in the astrocytes with Nef protein at 12 h, compared to 6 h (Fig. 4d).

We then analyzed the differential expression of lncRNAs in the astrocytes stimulated by Nef protein at 6 h and 12 h compared with 0 h, respectively. Results showed that 1358 lncRNAs were upregulated, and 670 lncRNAs were downregulated in the astrocytes stimulated by Nef protein for 6 h (Fig. 4e) and that 1534 upregulated and 1271 downregulated lncRNAs for 12 h (Fig. 4e). Notably, there were 638 co-upregulated and 372 co-downregulated lncRNAs for both 6 h and 12 h (Fig. 4f). The list of the top 20 differentially expressed lncRNAs for both 6 h and 12 h identified by microarray analysis was shown in Additional file 1: Table S2 and Table 2.

**Table 2:** Top 20 overlapped and upregulated lncRNAs in mouse astrocytes stimulated with Nef protein (50 ng/ml) for 6 h and 12 h

lncRNA seqname	Length	Upregulated folds	
		6 h/0 h	12 h/0 h
Gm16685	1592	33.808704	98.312874
Gm5970	1216	11.263321	75.315765
Gm12250	1274	6.2884264	61.74965
AK085771	1165	6.540899	66.45514
uc008roq.1	3961	45.042114	58.77
Gm4955	913	6.5966477	50.151352
A530040E14Rik	1262	10.544557	50.162678
Gm12407	720	6.1827254	33.648342
Gm13309	324	6.142315	30.465094
Mx2	2428	12.378684	37.599354
AK151815	1592	7.3327208	26.562384
MM9LINCRNAEXON11240-	1100	10.792236	20.239393
uc008smk.1	701	6.23503	32.482025
ENSMUST00000160565	1414	3.362174	22.119934
AK139352	4337	6.970084	36.466106
AK145170	2791	9.817351	20.934488
ENSMUST00000135659	1443	14.289103	18.727198
NR_003507	1848	3.7799745	14.091469
Gm8773	1268	5.3409157	10.783084
uc008zif.1	704	2.7248611	12.014651

### Validation of the microarray data using real-time PCR

**Fig. 5:**
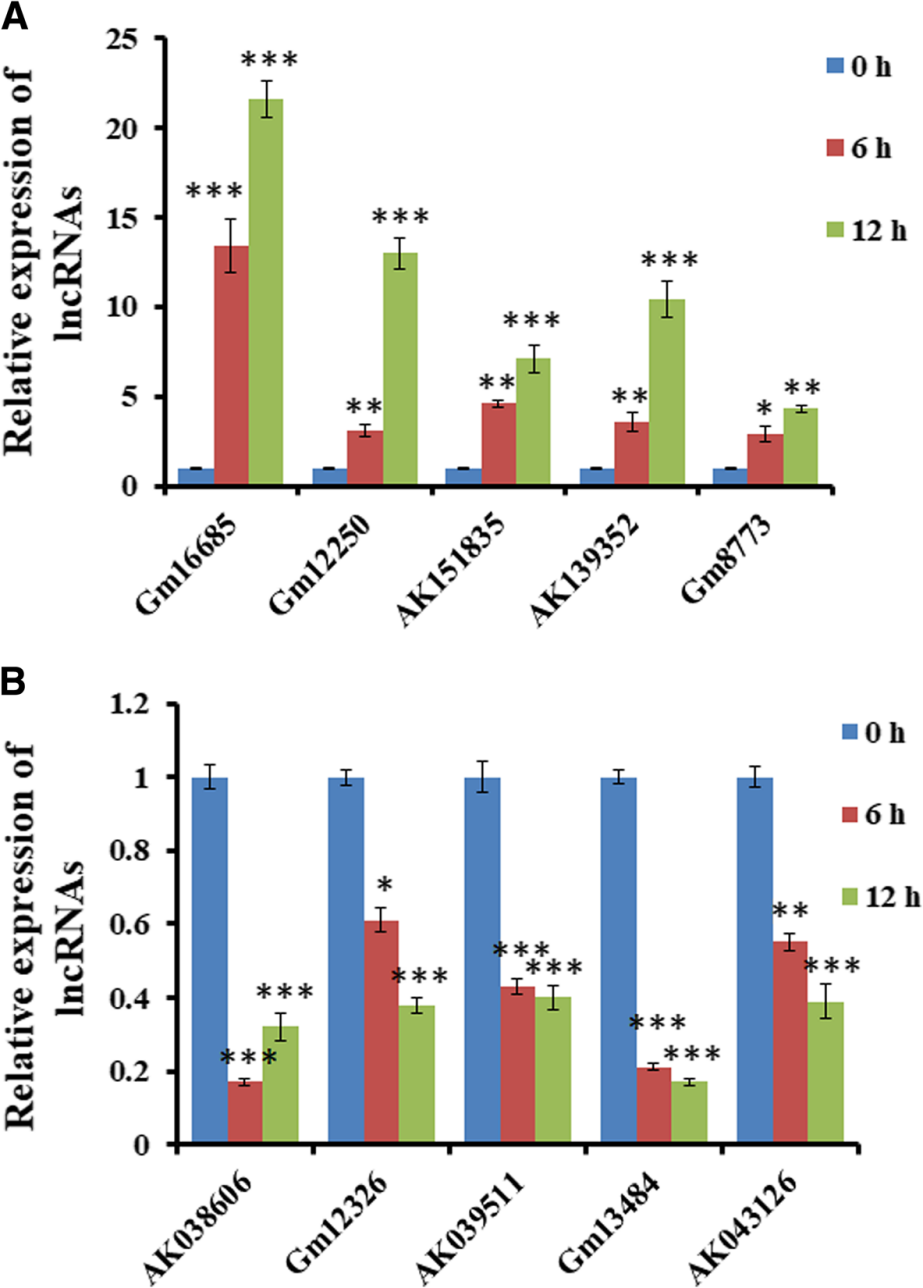
Real-time PCR verification of differentially co-expressed lncRNAs in astrocytes stimulated with Nef. Ten lncRNAs were randomly chosen for real-time PCR validation. **a** The expressions of lncRNA Gm16685, Gm12250, AK151815, AK139352, and Gm8773 were significantly increased in astrocytes stimulated with Nef protein (50 ng/ml) with 0 h, 6 h, and 12 h. **b** The expressions of lncRNA AK038606, Gm12326, AK039511, Gm13484, and AK043126 were markedly downregulated in astrocytes. *P < 0.05, **P < 0.01, ***P < 0.001, vs 0 h. Error bars represent means ± SEM. These data are from three independent experiments

To further verify the accuracy of microarray data, we randomly assigned 10 lncRNAs from the differentially expressed lncRNAs, including 5 co-upregulated (Gm16685, Gm12250, AK151815, AK139352, and Gm8773) and 5 co-downregulated (AK038606, Gm12326, AK039511, Gm13484, and AK043126) and tested their expression through real-time PCR assay. As shown in Fig. 5, Gm16685, Gm12250, AK151815, AK139352, and Gm8773 expression were significantly increased, whereas AK038606, Gm12326, AK039511, Gm13484, and AK043126 expression were obviously decreased in the astrocytes stimulated by Nef protein for 6 h and 12 h, compared with 0 h. Overall, these data confirmed the accuracy of microarray data.

### Analysis of upregulated lncRNAs on the chromosome 5 in the primary mouse astrocytes treated with Nef protein

**Fig. 6:**
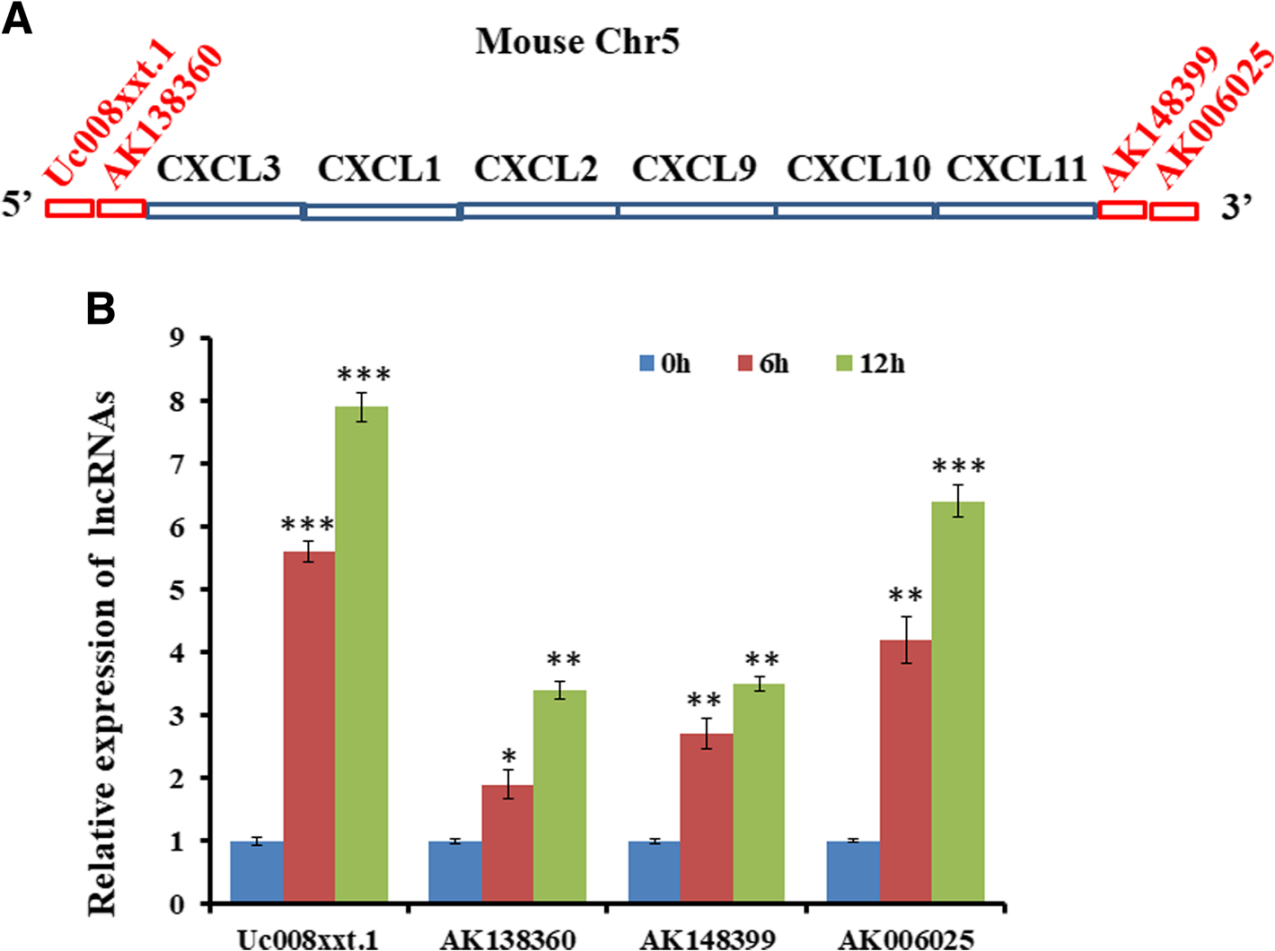
Verification of lncRNA location at two terminals of CXCL9/10/11 gene cluster on the mouse chromosome 5. **a** Schematic diagram of lncRNA location at two terminals of CXCL9/10/11 gene cluster. **b** Real-time PCR assay detection of lncRNA expression in astrocytes treated with Nef protein (50 ng/ml) with 0 h, 6 h, and 12 h. *P < 0.05, **P < 0.01, ***P < 0.001, vs 0 h. Error bars represent means ± SEM. These data are from three independent experiments

Then, we analyze differentially co-upregulated lncRNAs on chromosome 5 in the primary mouse astrocytes treated with Nef protein for 6 h and 12 h. As shown in Table 2, ten lncRNAs were selected as targeted ones. All of them were located at two terminals of Cxcl3/2/1 gene cluster and Cxcl/10/11 gene cluster (Additional file 1: Table S2). Among them, Uc008xxt.1 and AK138360 were close to 5′ terminal of Cxcl3/2/1 gene cluster while AK148399 and AK006025 were nearby the 3′ terminal of the Cxcl9/10/11 gene cluster (Fig. 6a). The class distribution of AK138360 and AK006025 were intergenic, Uc008xxt.1 was sense-overlap, and AK148399 was bidirectional (Additional file 1: Table S3).

We further measured above lncRNAs expression by real-time PCR in primary astrocytes stimulated with Nef protein. Results of real-time PCR showed that Uc008xxt.1, AK138360, AK148399, and AK006025 were significantly regulated (Fig. 6b), which was in accordance with the microarray data.

### Knockdown of lncRNA AK006025 repressed Cxcl9/10/11 cluster gene expression

**Fig. 7:**
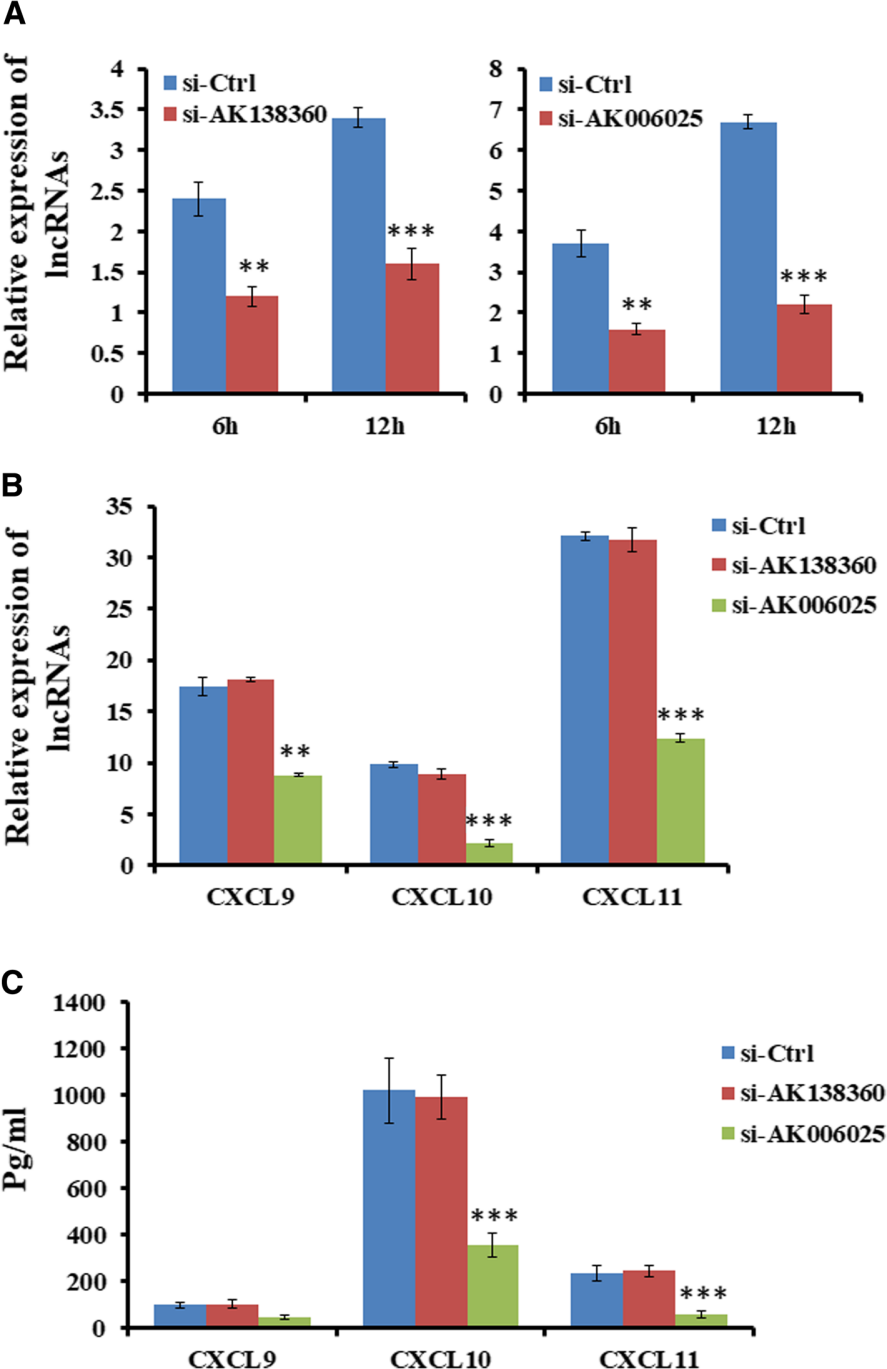
Knockdown of lncRNA AK006025 reduced CXCL9/10/11 cluster gene expression in astrocytes treated with Nef. **a** Real-time PCR assay detection of lncRNA expression in astrocytes transfected siRNAs for 48 h, followed by treatment with Nef protein for 6 h and 12 h. **b** Real-time PCR assay analysis of CXCL9, CXCL10, and CXCL11 mRNA transcriptional level in astrocytes transfected siRNAs for 48 h, followed by treatment with Nef protein for 12 h. **c** ELISA analysis of CXCL9, CXCL10, and CXCL11 secretion in astrocytes transfected siRNAs for 48 h, followed by treatment with Nef protein for 24 h. *P < 0.05, **P < 0.01, ***P < 0.001, vs 0 h. Error bars represent means ± SEM. These data are from three independent experiments

Intergenic lncRNA, as an in *cis*-regulatory element, regulates gene expression. To determine the role of intergenic lncRNAs in regulating Cxcl9/10/11 cluster gene expression, siRNA of AK138360 or AK006025 was transfected into primary mouse astrocytes for 48 h, followed by Nef protein treatment for 6 h and 12 h, respectively. We detected Cxcl9/10/11 expression using ELISA and real-time PCR. As seen from Fig. 7a, siRNA of AK138360 or AK006025 inhibited their expression. All of Cxcl9, Cxcl10, and Cxcl11 expression were significantly decreased in mouse astrocytes treated with Nef protein after knockdown of AK006025, not AK138360 (Fig. 7b).

### AK006025 associated with CBP/P300 regulates Nef-induced Cxcl9/10/11 cluster gene expression

**Fig. 8:**
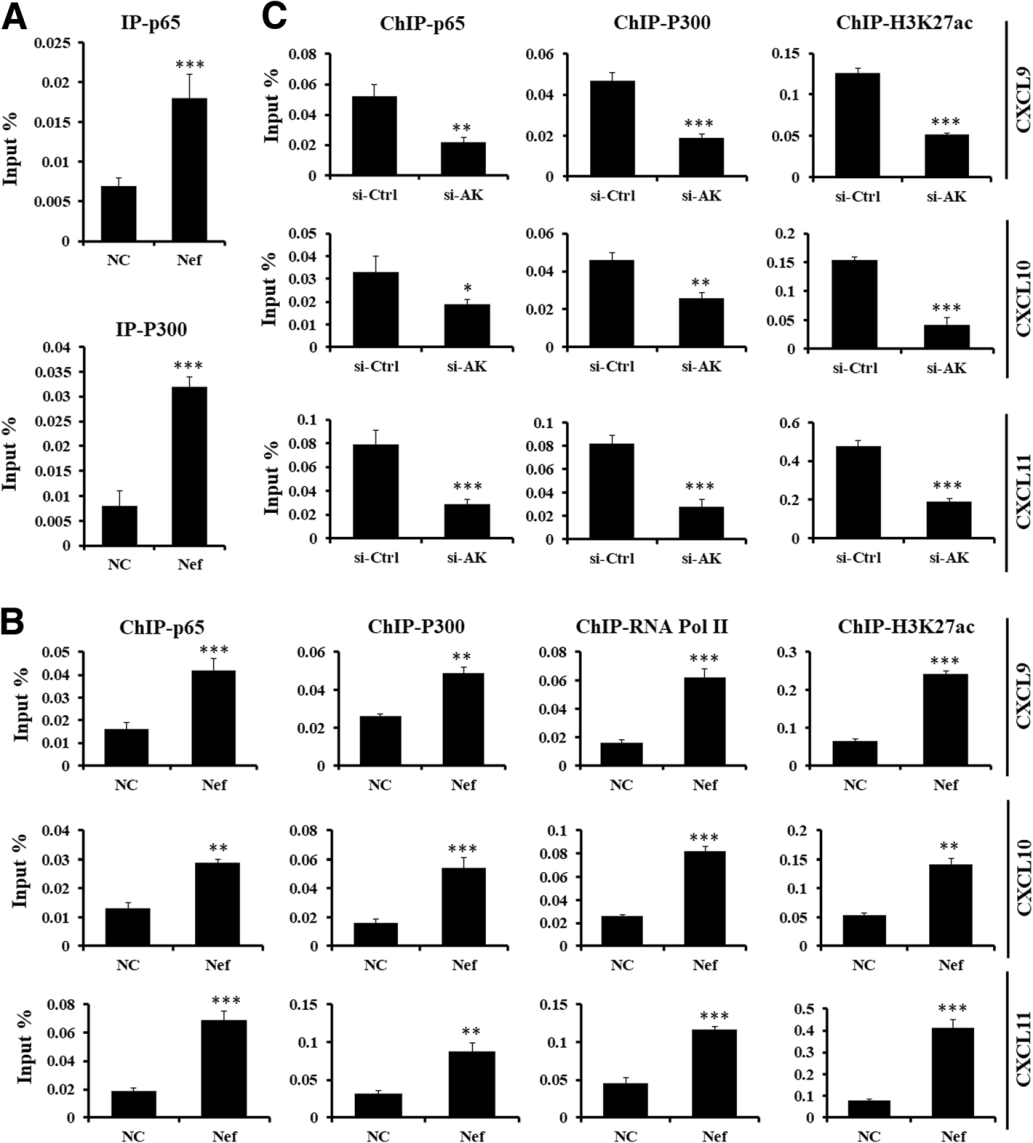
Analysis of lncRNA AK006025 interaction with CBP/P300 to regulate CXCL9/10/11 cluster gene expression in astrocytes treated with Nef. **a** RIP assay analysis of the interaction of AK006025 with NF-κB p65 and CBP/P300 in astrocytes treatment with Nef protein for 3 h. NC: natural control. ***P < 0.001, vs NC. These data are from three independent experiments. **b** ChIP assay analysis of NF-κB p65, CBP/P300, RNA Pol II, and H3K27ac enrichment on the promoter of CXCL9/10/11 gene cluster in astrocytes treatment with Nef protein for 3 h. **P < 0.01, ***P < 0.001, vs NC. Error bars represent means ± SEM. These data are from three independent experiments. **c** ChIP assay analysis of NF-κB p65, CBP/P300, and H3K27ac enrichment on the promoter of CXCL9/10/11 gene cluster in astrocytes transfected siRNAs for 48 h, followed by treatment with Nef protein for 3 h. si-AK: si-AK006025. *P < 0.05, **P < 0.01, ***P < 0.001, vs his-Ctrl. Error bars represent means ± SEM. These data are from three independent experiments

To further uncover the potential mechanism how lncRNA AK006025 regulated Cxcl9/10/11 cluster gene expression, RIP assay was used for detecting lncRNA AK006025 and NF-κB p65 and/or CBP/P300 interaction. RIP results showed that AK006025 was significantly associated with NF-κB p65 and CBP/P300 in mouse astrocytes treated with Nef protein for 3 h (Fig. 8a). Furthermore, ChIP assay indicated that NF-κB p65, CBP/P300, RNA Pol II, and H3K27ac were differentially enriched in in the promoter Cxcl9/10/11 gene cluster in mouse astrocytes treated with Nef protein for 3 h (Fig. 8b). Of importance, siRNA of AK006025 repressed dramatically NF-κB p65 and CBP/P300 enrichment in the promoter Cxcl9/10/11 gene cluster in mouse astrocytes treated with Nef protein for 3 h (Fig. 8c). The data suggest that the interaction of AK006025 and CBP/P300 may regulate epigenetically Cxcl9/10/11 cluster gene transcription.

## Discussion

HAND is a degenerative disease of the central nervous system with chronic inflammation, synapse dysfunction, and neuron loss. Many studies have determined that the blockade of pro-inflammatory cytokine and chemokine production in astrocytes could protect the neuron damage of HAND pathogenesis [13, 16, 17].

Nef, a myristoylated protein encoded by HIV-1, functions in the multifaceted biological process including trafficking, signal transduction, and gene expression in a paracrine manner. Nef is released from infected cells into the plasma of HIV-infected individuals [53]. The detectable concentration of soluble Nef in the serum ranges from 1 to 10 ng/ml [54, 55]. The soluble Nef can be taken up by several types of cells to regulate cellular function, such as B cells, primary effusion lymphoma (PEL) cells, pulmonary arterial endothelial cells as well as primary human umbilical vein endothelial cells (HUVECs) [56–59]. Although HIV-1 built latency in astrocytes in the brain, the expression of HIV-1 genes are low, for example, HIV p24 in HIV-1 ^+^ postmortem brain astrocytes is rarely detected [60]. Therefore, we used soluble Nef protein as an exogenous factor to explore its role in inducing the expression of lncRNAs and mRNAs. Here, our data showed that the exogenous Nef protein enhanced CCL5 and IP-10 expression in astrocytes, consistent with previous data of endogenous Nef expression [16, 25].

Over the past decades, the molecular mechanisms underlying HAND have been extensively explored. However, the understanding of the pathophysiological process of HAND is still limited. In recent years, a large number of evidence shows that lncRNAs have been associated with human diseases, such as cancer, neurological disorders, and so on [30, 31, 52, 61]. LncRNAs are found as important regulators of neurodevelopment and brain function [31]. However, there is no knowledge that lncRNAs are associated with a particular molecular or cellular function in HAND pathogenesis. To date, the functional characterization of lncRNAs during the HAND/HAD process and astrocyte activation has not been uncovered. Our recent study demonstrated the expression profiles of lncRNAs in Nef-treated astrocytes, analyzed the co-expression of lncRNAs in vivo and in vitro, and explored their characteristics and possible relations with protein-coding genes.

In our study, we found that there were 1385 upregulated lncRNAs and 734 upregulated mRNAs in the astrocytes stimulated by Nef protein for 6 h; meanwhile, 670 lncRNAs and 876 mRNAs were downregulated. Furthermore, 1534 lncRNAs and 1077 mRNAs were upregulated, and 1271 lncRNAs and 982 mRNAs were downregulated in the astrocytes stimulated by Nef for 6 h. Of importance, there were 638 lncRNAs and 460 mRNAs that differentially co-upregulated, and 372 lncRNAs and 376 mRNAs that differentially co-downregulated in the astrocytes treated with Nef for both 6 h and 12 h. Moreover, real-time PCR assay was performed to confirm parts of differentially expressed lncRNAs and mRNAs, which was consistent with the results of lncRNAs microarray. Interestingly, the differentially-expressed lncRNAs appear to have a relationship with the levels of inflammatory cytokines and chemokine secreted by astrocytes, suggesting that these lncRNAs might uncover novel insight into the molecular basis of HAND pathogenesis.

In our studies, a large number of inflammatory genes were significantly co-upregulated in the astrocytes treated with Nef for both 6 h and 12 h, especially *Cxcl11*, *Cxcl10*, *Cxcl9*, *Cxcl3*, *Cxcl2*, *Cxcl1*, *Ccl5*, *Il-1β*, *Il-6*, and *Tnf.* Furthermore, some mRNAs including *Ifnb1*, *Irg1*, *Tnfsf10*, and *CD69* were also screened out and dramatically increased (Table 1), whose functions are associated with immune response, interferon (IFN) signal as well as apoptosis. Therefore, further studies are needed to demonstrate whether they are involved in HAND process.

CXC chemokine, structurally recognizable by the position of four conserved cysteine residues, are prominent mediators of chemotaxis. The Cxcl9/10/11 genes locate on the mouse chromosome 5 in cluster manner and are induced by IFN [62]. Previous data showed that Nef increased IP-10 expression in astrocytes [25]. Here, we scanned the chemokine expression profile in primary mouse astrocytes treated with Nef protein. The result showed that Cxcl9, Cxcl10, and Cxcl11 were all top differentially expressed; of importance, Nef increased CXCL9, CXCL10, and CXCL11, mRNA levels in human astrocytes, which suggests an important role in HAND pathogenesis.

Emerging data show that lncRNAs are involved in regulating NF-κB signaling, anti-viral response, and inflammatory response [36–39, 47]. In the present study, based on the GO term enrichment and pathway maps of mRNAs, we found that markedly enriched molecular functions and biological processes of upregulated genes in astrocytes were mainly associated with cytokine and chemokine activity as well as their receptor binding in astrocytes treated with Nef protein. These findings are consistent with previous data showing that the infiltration of immune cells and inflammation play a vital role in the pathogenesis of HAND [17].

It has been established that lncRNAs have been shown to link a number of epigenetic modified complexes with transcriptional factors including NF-κB, resulting in regulating gene expression [33, 34, 36]. In addition, lncRNAs bind CBP/P300 activity to regulate gene expression [47]. Here, we analyzed four lncRNAs located on 5′ terminal and 3′ terminal of Cxcl3/2/1 gene cluster and Cxcl9/10/11 gene cluster. Among them, AK138360 and AK006025 siRNA located on 3′ terminal of the Cxcl9/10/11 gene cluster. Unexpectedly, all of Cxcl9, Cxcl10, and Cxcl11 expression were dramatically decreased in mouse astrocytes treated with Nef protein after knockdown of AK006025, not AK138360. Furthermore, RIP and ChIP assay showed that AK006025 associated with NF-κB p65 and CBP/P300 was enriched in the promoter of Cxcl9, Cxcl10, and Cxcl11. These data suggest that interaction of AK006025 and CBP/P300 might regulate epigenetically Cxcl9/10/11 cluster gene transcription in HAND pathogenesis.

So far, our findings still have several limitations. First of all, these studies only were performed to scan lncRNA profiles in primary mouse astrocytes, which is possibly different from that of human astrocytes, particularly for ncRNAs that are heterogeneous expressions in mouse cells. Secondly, we only determined the role of a single acute exposure to Nef protein, which may differ from the effects of chronic exposure in HAND pathogenesis. Therefore, it is important to demonstrate how Nef influences lncRNAs expression in human astrocytes in HAND.

## Conclusion

In summary, our results revealed that Nef induced thousands of differentially expressed lncRNAs in astrocytes. LincRNA AK006025 was involved in regulating Nef-induced CXCL9, CXCL10, and CXCL11 expression through interaction with NF-κB p65 and CBP/P300, which may play key roles in neuroinflammation and pathogenesis of HAND.

## Additional file

Additional file 1: **Table S1.** Primer sequences for real-time PCR assay. **Table S2.** Top 20 overlapped and downregulated lncRNAs in mouse astrocytes stimulated with Nef protein (50 ng/ml) for 6 h and 12 h. **Table S3.** Analysis of differentially upregulated lncRNAs on the chromosome 5 in the mouse astrocytes stimulated with Nef protein (50 ng/ml) for 6 h and 12 h. (DOC 118 kb)

## Abbreviations

AIDS: Acquired immunodeficiency syndrome
BBB: Blood-brain barrier
CBP/P300: CREB-binding protein
ChIP: Chromatin immunoprecipitation
CNS: Central nervous system
GFAP: Glial fibrillary acidic protein
GO: Gene ontology
HAART: Highly active antiretroviral therapy
HAD: HIV-associated dementia
HAND: HIV-associated neurocognitive disorder
HDAC1: Histone deacetylase 1
HIV-1: Human immunodeficiency virus type 1
IL-1β: Interleukin-1 betta
IP-10: Interferon-gamma-inducible 10-Kd protein
lncRNAs: Long non-coding RNAs
MF: Molecular function
Nef: Negative factor
NF-κB: Nuclear factor-kappa B
RIP: RNA immunoprecipitation
Tat: Transactivator of transcription
TNF: Tumor necrosis factor
