# Supplementary material for: HIV-1 Nef-induced lncRNA AK006025 regulates CXCL9/10/11 cluster gene expression in astrocytes through interaction with CBP/P300
Source: J Neuroinflammation. 2018 Oct 31;15:303. doi: 10.1186/s12974-018-1343-x (PMC6208068; doi:10.1186/s12974-018-1343-x)
Supplement: Supplementary file 1 — Table S1. Primer sequences for real-time PCR assay. Table S2. Top 20 overlapped and downregulated lncRNAs in mouse astrocytes stimulated with Nef protein (50 ng/ml) for 6 h and 12 h. Table S3. Analysis of differentially upregulated lncRNAs on the chromosome 5 in the mouse astrocytes stimulated with Nef protein (50 ng/ml) for 6 h and 12 h. (DOC 118 kb) [file 12974_2018_1343_MOESM1_ESM.doc]

**Tables**

**Table S1. Primer sequences for qPCR.**

| **Primer Name** | **Sequence (5’ to 3’)** | **Length (bp)** |
| --- | --- | --- |
| **Primers for lncRNAs** |  |  |
| Gm16685 | Fw GCCCTACAGGAAGACCAAC | 174 |
|  | Rev GAGCCTTCACAGGACCAA |  |
| Gm12250 | Fw CTGGAGGTGATCCGAAGTG | 193 |
|  | Rev CAGGCTCAGTAATGGTAGGG |  |
| AK151815 | Fw GCCCTACAGGAAGACCAAC | 128 |
|  | Rev AGGCCATCCTCTGCTACG |  |
| AK139352 | Fw ACAACGCTGAGATTCCACC | 159 |
|  | Rev GAACCGCCAGACTGATTT |  |
| Gm8773 | Fw ACTGCCTACTGCCAACACG | 192 |
|  | Rev CAGGACCTCGCTCCTTTT |  |
| AK038606 | Fw ATGGTAAACATCCCAAGCC | 179 |
|  | Rev GAGGTCCACATGATTTCTTCCT |  |
| Gm12326 | Fw ACAGGATTATGTATGAGGCAGAG | 174 |
|  | Rev CCAGGCTAATGAAAGCAAGA |  |
| AK039511 | Fw TCCTTGCCTCCACTTCCT | 196 |
|  | Rev ATGCCACTGATAATCTTACAACC |  |
| Gm13484 | Fw TTAGGCAATGGCACAGTTT | 146 |
|  | Rev CTTCTCGGTACATAGGCTCAC |  |
| AK043126 | Fw AAAGGCATCTAAGGAGTGG | 173 |
|  | Rev AAGGGAGTGTTGGACCTG |  |
| Uc008xxt.1 | Fw AGAAGGGTTGGAGGGAAGA | 169 |
|  | Rev TACTGGGCGTTGAATGGA |  |
| AK138360 | Fw TAGCCAGTAGGCAGAGGG | 188 |
|  | Rev GCAATGAAGCGCAAATCC |  |
| AK148399 | Fw TCGCTTACTACCACGCTTTCTATG | 134 |
|  | Rev GGGATGAAGGCTTTCCACTA |  |
| AK006025 | Fw CCTGAACGCCTCCAAATC | 178 |
|  | Rev TCAGAGCAGCAGCAACCA |  |
|  |  |  |
| **Primers for mRNAs** |  |  |
| Mouse Cxcl9 | Fw CATACACCCATAATCTCAGACG | 143 |
|  | Rev GAGCAGTTTCCAGCTCCC |  |
| Mouse Cxcl10 | Fw ATTGCCCTTGGTCTTCTGA | 197 |
|  | Rev CGCACCTCCACATAGCTTAC |  |
| Mouse Cxcl11 | Fw CAAGCAAGCTCGCCTCAT | 153 |
|  | Rev TGGCTGCATGTTCCAAGAC |  |
|  |  |  |
| Human CXCL9 | Fw CATCTTGCTGGTTCTGATTGGAGTG |  |
|  | Rev GATAGTCCCTTGGTTGGTGCTGAT |  |
| Human CXCL10 | Fw GTGGCATTCAAGGAGTACCTC |  |
|  | Rev TGATGGCCTTCGATTCTGGATT |  |
| Human CXCL11 | Fw TGTGCTACAGTTGTTCAAGGCTTCC |  |
|  | Rev GCTTGCTTCGATTTGGGATTTAGGC |  |
| Actin | Fw CATGTACGTTGCTATCCAGGC |  |
|  | Rev CTCCTTAATGTCACGCACGAT |  |
|  |  |  |
| **Primers for RIP** |  |  |
| AK0060250-1 | Fw TTCCTGAACGCCTCCAAA | 180 |
|  | Rev TCAGAGCAGCAGCAACCA |  |
| AK006025-2 | Fw CCAGCTCGTCCGAATCCT | 130 |
|  | Rev AATGCAGGTGCCCTCAAA |  |
| AK006025-3 | Fw ACTGTACTAACTACCACCTGAGAC | 144 |
|  | Rev GGCCAGCTCTTTCTACAATCT |  |
|  |  |  |
| **Primers for ChIP** |  |  |
| Cxcl9-1 | Fw TCACATCCCTTACTATAAACTCC | 151 |
|  | Rev CTGATTGGCTGGTCTCCT |  |
| Cxcl9-2 | Fw CACGGTGAGACGAAATGG | 179 |
|  | Rev GCAAAGTTGCTACCTGGAT |  |
| Cxcl9-3 | Fw CCAGGACGATCAATTTGTG | 143 |
|  | Rev AGTGAAGTCCGAGAATGTCT |  |
| Cxcl9-4 | Fw ACATCCCTTACTATAAACTCCC | 147 |
|  | Rev GATTGGCTGGTCTCCTCA |  |
| Cxcl10-1 | Fw AACAGCTCACGCTTTGGA | 182 |
|  | Rev TGATTGGCTGACTTTGGAG |  |
| Cxcl10-2 | Fw GGCACTGCATCTGATTTCT | 141 |
|  | Rev ACCGAGGGCATTGCTTGT |  |
| Cxcl10-3 | Fw AAGCAATGCCCTCGGTTTA | 157 |
|  | Rev CTCAGCGGTGGATGAAGC |  |
| Cxcl10-4 | Fw TCCAAGTTCATGGGTCACAA | 137 |
|  | Rev TTTATCTGCAAAGAGTTTCCCT |  |
| Cxcl11-1 | Fw TGAGGGAGGCAGGATGAG | 145 |
|  | Rev AAGCCACTGGAAGGTGAAA |  |
| Cxcl11-2 | Fw AGTTGGTGGGACTCTGCC | 177 |
|  | Rev CGTAGCTTTCTTGCCTCCTG |  |
| Cxcl11-3 | Fw ATGGCGTCTCAGAATGGC | 158 |
|  | Rev AGATGGCACAGGAGGTTGTT |  |
| Cxcl11-4 | Fw ACTTTAAGAATACGCTGGAACAAC | 150 |
|  | Rev CAGTAAGGAAGAGGAGGAGGC |  |

**Table S2. Top 20 overlapped and down-regulated lncRNAs in mouse astrocytes stimulated with Nef protein (50 ng/ml) for 6 h and 12 h**

| **lncRNA seqname** | **length** | **down-regulated folds** | |
| --- | --- | --- | --- |
|  |  | **6 h/0 h** | **12 h/0 h** |
| AK038606 | 1031 | -20.922466 | -11.259366 |
| AK021366 | 393 | -4.8497653 | -10.662202 |
| Gm13484 | 412 | -12.615703 | -17.251356 |
| AK028578 | 3285 | -9.13495 | -11.743698 |
| Gm12326 | 618 | -3.7615118 | -11.638856 |
| AK133261 | 2582 | -9.1141 | -11.303252 |
| ENSMUST00000128165 | 2116 | -2.8536913 | -9.596995 |
| MM9LINCRNAEXON10468+ | 229 | -6.0239553 | -8.006237 |
| Gm14589 | 727 | -9.038001 | -8.337769 |
| 1700045I19Rik | 1190 | -4.1116533 | -8.990601 |
| Gm16226 | 354 | -10.431743 | -9.679331 |
| mouselincRNA1514- | 10077 | -9.112503 | -9.258471 |
| mouselincRNA0780 | 387 | -2.5779457 | -7.2771873 |
| AK045794 | 3817 | -6.3484974 | -7.504542 |
| AK039511 | 918 | -7.323936 | -7.5077195 |
| ENSMUST00000167632 | 353 | -4.609916 | -7.127153 |
| AK081799 | 1686 | -5.0048623 | -6.8012457 |
| AK020176 | 914 | -4.0325155 | -6.752369 |
| ENSMUST00000146582 | 1780 | -4.592875 | -6.4143114 |
| AK043126 | 2190 | -2.6642187 | -9.983197 |

**Table S3. Differentially up-regulated lncRNAs on the chromosome 5 in the mouse astrocytes stimulated with Nef protein (50 ng/ml) for 6 h and 12 h**

| **lncRNA seqname** | **length** | **strand** | **txStart** | **txEnd** | **Class distribution** |
| --- | --- | --- | --- | --- | --- |
| uc008zif.1 | 704 | + | 121264425 | 121271435 | sense overlap |
| NR_003507 | 1848 | + | 121262643 | 121274169 | sense overlap |
| Gm8773 | 1268 | + | 5573798 | 5576203 | intergenic |
| uc008xxt.1 | 3123 | - | 86992079 | 87061449 | sense overlap |
| AK037802 | 1295 | - | 69961431 | 69962722 | antisense overlap |
| AK006025 | 656 | + | 97278523 | 97281957 | intergenic |
| AK138360 | 701 | + | 90019790 | 90020490 | intergenic |
| Gm3414 | 7912 | - | 46110905 | 46118817 | sense overlap |
| ENSMUST00000170544 | 1833 | + | 110814853 | 110817092 | antisense overlap |
| AK148399 | 2139 | - | 93520019 | 93522156 | bidirectional |
| AK089560 | 2683 | + | 13525726 | 13528408 | sense overlap |
